# Dr. Bryan on Prophylaxis and Criticisms

**Published:** 1904-01

**Authors:** L. C. Bryan

**Affiliations:** Basel, Switzerland


### Foreign Correspondence.

### DR. BRYAN ON PROPHYLAXIS AND CRITICISMS.

Basel, Switzerland, November 19, 1903.

To the Editor:

Sir,—In the discussion on my article on "Prophylaxis," in the November, 1903, number of your journal, page 831, reported from The New York Institute of Stomatology, on page 854, I

notice with pleasure that there is a growing sentiment in America in favor of these thorough operations which *prevent decay* of the teeth. I also note that there is a misconception of the conditions existing in Europe *versus* those in America. In America, dentistry is sixty years ahead, so far as the public is concerned, in the personal care of the teeth, and with the American patients that I occasionally see there is a decided difference in the way they take care of their teeth and the way representatives of other nations treat theirs. No wonder decay is not on the increase in America. In Basel we have had a long series of American dentists who have trained the people of the upper class in certain things, but still when an American lady comes to me I can tell her teeth from those of any other nation, simply by the way they are treated prophylactically by her (I am not so sure of the masculines). They are *clean,* they are polished like ivory, they have good gold and other fillings, the teeth shine like alabaster. It is a joy and a pleasure to see them, and do the little that is to be done on them, and one has the feeling that the work one does will prove a success for years, and it is a stimulation to *do* good work. One knows that they will brush their teeth as only Americans know how to brush them; that the waxed silk, rinsing, and brushing will remove every particle of food, every night before the patient retires; that they will adopt every suggestion of the dentist, if they do not already know more of the personal care of the teeth than the occasional dentist will tell them between the acts; that they will go to their dentist regularly and never neglect their teeth till they "feel a pain;" in fact, that they will love their dentist and keep his commandments. I do not know whether I should thank the Almighty that I have been sent abroad as a missionary in dentistry or regret the Providence that has removed me from my own people, who are such model patients to prophylaxis or personal care of their teeth.

The above will be sufficient answer to Drs. Gillett and Kimball's criticism of the first paragraph of my paper. I do not have difficulty in doing the "little things" that go to make up the hours of work charged under the heading of "various operations." My people know what they get, and know that these "little things," these "various operations," will prevent more "tedious and expensive operations" in coming years. They already see that the "preventive dentistry" saves them, and especially their children, hours of agony and great expense that they have gone through themselves in former years, when the slightest defect or sensitive superficial decay, that is now stopped with preventive remedies, was bored out with pain and filled with expense. This naturally brings in their friends who have had such a dread that they have never dared to go to a dentist, and their teeth have been left to bacteria and the forceps, under the benign influence of gas, until they need bridge-work or a wabbly plate, which latter should be as much a shame and embarrassment (and usually is) as a wooden leg or a rubber nose.

Dr. F. Milton Smith says "there is nothing new in either the paper of Dr. Bryan or that by Dr. Smith, of Philadelphia." Well, it would be something new if a Dr. Smith of New York would acknowledge that a Dr. Smith of Philadelphia could tell him anything. I have looked pretty carefully for some remedy which was recommended to be applied externally to weak, newly erupted teeth to prevent decay, and have not found it in medical or dental literature for the last fifty years, and I believe this *is* something new, and I believe I have for the Dental Congress in St. Louis some other new things in this line, and will present them there. As for "Dr. Smith of Philadelphia," the profession had better keep an eye on his methods, and also give just a little time to consideration of Dr. Wright's (Cincinnati) suggestion of training feminine assistants to do this prophylactic work, and then you will see not only the Americans who travel abroad, with teeth like pearls, but those who stay at home may gladden the hearts of those dentists who appreciate a clean laboratory,—the human mouth.

Yes, we had polishing with wood points in porte-polishers a generation and more ago, and bathing in the Jordan was recommended long before many Saratogas and Hot Springs were known, and still it's a good thing to recommend the latter nowadays, and it's a new thing under the Western sun.

With greetings to the home brethren, I am yours, fraternally,

L. C. Bryan.

